# Pulmonary Malignancies in Adults With Congenital Lung Malformations: A Scoping Review

**DOI:** 10.1016/j.jtocrr.2026.100958

**Published:** 2026-01-16

**Authors:** Louis W.J.R. Dossche, M.H.T. Heuff, A.M. Heijne den Bak, A.C. Dingemans, L.S. Kamphuis, J. Marco Schnater

**Affiliations:** a Department of Pediatric Surgery, Erasmus MC Sophia Children’s Hospital, University Medical Center Rotterdam, Rotterdam, The Netherlands; b Department of Pulmonology, Erasmus MC, University Medical Center Rotterdam, Rotterdam, The Netherlands

**Keywords:** Congenital lung malformations, Congenital pulmonary airway malformation, NSCLC, Adenocarcinoma, Thoracic oncology, Pulmonary malignancy

## Abstract

We aimed to synthesize current knowledge on pulmonary malignancies in adults with congenital lung malformations (CLMs), focusing on clinical presentation, treatment approaches, and outcomes.

We performed a scoping review according to the Preferred Reporting Items for Systematic Reviews and Meta-Analyses extension for scoping reviews. A systematic search was conducted in January 2025 across three databases. References were included if they described patients aged 17 years or older with a CLM and a coexisting pulmonary malignancy. Data were extracted on patient demographics, CLM and malignancy type, symptoms, treatment, and outcomes. Findings were summarized using descriptive statistics and narrative synthesis.

A total of 65 references met the inclusion criteria, comprising 85 adult patients with CLM and pulmonary malignancies. Pulmonary adenocarcinoma was most frequently observed (66%), particularly within congenital pulmonary airway malformations (89% of malignancies in that subgroup). The median age at lung cancer diagnosis was 43.5 years. Respiratory symptoms were the most common presentation (67%), with 18% of patients being asymptomatic at the time of pulmonary malignancy diagnosis. Surgical resection was performed in 94% of patients, with lobectomy being the most frequently performed surgery type (68% of operated patients). More than half of malignancies (57%) were definitively diagnosed postoperatively. Follow-up data were incomplete; however, most reported patients were alive without disease recurrence at last follow-up with a median follow-up time of 14 months.

The young median age at cancer diagnosis, the presence of pulmonary malignancies in asymptomatic patients, and the high proportion of malignancy diagnoses established only postoperatively underscore the need for proactive and tailored surveillance strategies in adults with known CLM. A multidisciplinary approach with longitudinal follow-up is crucial to refine risk assessment and optimize long-term care.

## Introduction

Congenital lung malformations (CLMs) encompass a spectrum of rare developmental anomalies affecting the respiratory tract, including congenital pulmonary airway malformation (CPAM), bronchopulmonary sequestration (BPS), bronchogenic cyst (BC), congenital lobar overinflation (CLO), bronchial atresia, and hybrid lesions that typically combine features of CPAM and BPS.^1^^,^^2^ The estimated incidence of CLM is approximately 4 per 10,000 live births, with an apparent rise in reported cases in recent decades, attributed in part to the widespread use of routine prenatal ultrasound screening and advancements in ultrasound imaging resolution.^3^, ^4^, ^5^

Up to 80% of patients with CLM are asymptomatic at birth, and only a few experience clinical manifestations during childhood, such as respiratory distress, pneumothorax, cardiac overload, and recurrent respiratory infections.^6^, ^7^, ^8^ The optimal management of asymptomatic patients remains a topic of debate and is currently being investigated in the COllaborative Neonatal Network for the first European CPAM Trial (ClinicalTrials.gov ID NCT05701514).^9^^,^^10^ Some clinicians advocate for a conservative approach, emphasizing the substantial proportion of patients who remain symptom free and cautioning against overtreatment.^11^ Conversely, others favor prophylactic surgery because of the risk of malignant transformation, a rare yet potentially lethal phenomenon with a poorly understood etiology.^9^ Estimating the incidence of malignant transformation in adulthood is currently impossible as the denominator—the prevalence of CLM in adults—remains unknown.

Research on malignant transformation in patients with CLM is limited, particularly in adult populations. Most existing studies focus on pediatric patients, especially those involving CPAM, where malignant transformation is often associated with specific tumor types, such as pleuropulmonary blastoma.^12^ Recent research has explored molecular pathways involved in malignant transformation, identifying genomic instability and mutations in oncogenes as potential contributors.^13^^,^^14^ In adults with CLM, the clinical evidence is primarily restricted to case reports and small case series, which individually provide limited insight into the patterns of clinical presentation, management approaches, and outcomes in this population.

This scoping review aims to consolidate current knowledge on pulmonary malignancies in adults with CLM, providing an overview of clinical characteristics, treatment approaches, and patient outcomes. The guiding research questions for this review are the following:

(1) What types of pulmonary malignancy are most common in adults with CLM?

(2a) How and (2b) at what age do pulmonary malignancies typically present in adults with CLM?

(3) What are common treatment approaches for pulmonary malignancies in adults with CLM?

## Methods

This scoping review was performed according to the Preferred Reporting Items for Systematic Reviews and Meta-Analyses extension for scoping reviews,^15^ with a completed checklist provided as Supplementary File 1. A predefined study outline was registered on the Open Science Framework on October 2, 2024, and is publicly accessible at https://osf.io/4hp3b.

### Eligibility Criteria

References were included if they reported on patients with CLM aged 17 years or older diagnosed with a pulmonary malignancy. We defined CLM as any of the following anomalies: CPAM, BPS, CLO, BC, bronchial atresia, or hybrid lesions. Eligible publication types included case reports, case series, cohort studies, conference abstracts, and clinical reviews, provided that full text was available in English and contained individual patient-level data. Editorials, comments, and retracted articles were excluded from the results.

### Search

A systematic search strategy was developed by a biomedical information specialist from the medical library at our center. Searches were conducted in January 2025 across the following three databases: MEDLINE, Embase, and Web of Science. Detailed electronic search queries for each database are provided in Supplementary File 2.

### Study Selection

After removing duplicates, references were independently screened by two reviewers (L.W.J.D. and M.H.T.H.) based on the title, abstract, and keywords. Both reviewers conducted the initial screening separately and discussed any discrepancies until consensus was reached. Full-text articles were independently evaluated by the same reviewers against the predefined inclusion and exclusion criteria. In cases of disagreement during the full-text assessment, differences were resolved through discussion to ensure accurate and consistent inclusion of relevant evidence.

### Data Charting Process

A data-charting form was jointly developed by L.W.J.D. and M.H.T.H. within Covidence, an online systematic review management platform. The form captured key details such as author name, region of origin, year of publication, patient demographics, and clinical data (further detailed under “Data Items”). Before full implementation, the form was tested by both reviewers to ensure comprehensive data capture. Adjustments were made during this calibration exercise to refine the form and improve clarity.

### Data Items

The demographic characteristics of interest were sex and age at the time of cancer diagnosis. The clinical data collected included the type of CLM, the type of pulmonary malignancy, smoking status, symptoms leading to the discovery of the malignancy, the administered treatment, and the patient follow-up status.

To facilitate clearer comparisons, several clinical characteristics were categorized. First, the type of CLM was categorized as CPAM, intralobar BPS (ILBPS), extralobar BPS, BPS not further specified, BC, CLO, or bronchial atresia. Second, the malignancy type was categorized into adenocarcinoma (including mucinous, mixed, formerly called bronchoalveolar carcinoma, and not further specified), carcinoid, other carcinoma forms, and other non-carcinoma malignancies. To minimize interpretational errors, we recorded tumor staging data only when primary references explicitly mentioned this information according to the *TNM Classification of Malignant Tumours* format.^16^ Third, symptoms were grouped into respiratory symptoms (including cough, dyspnea, respiratory tract or lesion infections, hemoptysis), systemic symptoms (including fever, weight loss, asthenia), pain, and other. Last, treatment approaches were categorized as resection (segmentectomy, lobectomy, pneumonectomy, non-anatomical, or other), lymph node dissection, chemotherapy, radiotherapy, or other.

### Critical Appraisal of Evidence

Consistent with the standard methodology for scoping reviews, a formal critical appraisal of the included sources was not conducted. The primary objective of this scoping review was to provide a comprehensive overview of the existing literature on pulmonary malignancies in adults with CLM, irrespective of the methodological quality or potential risk of bias in the individual studies.

### Synthesis of Results

Data synthesis and presentation were performed using a combination of narrative summaries and detailed tables. This dual approach—combining visual, structured tables and descriptive narratives—ensures a comprehensive and clear presentation of the data, making it easier to identify potential relationships between variables and gain insights into the clinical characteristics of adults with CLM and pulmonary malignancies. The statistical package IBM SPSS (version 28.0.1.0) was used for data analysis.

## Results

### Reference Selection and Characteristics

**Figure 1:**
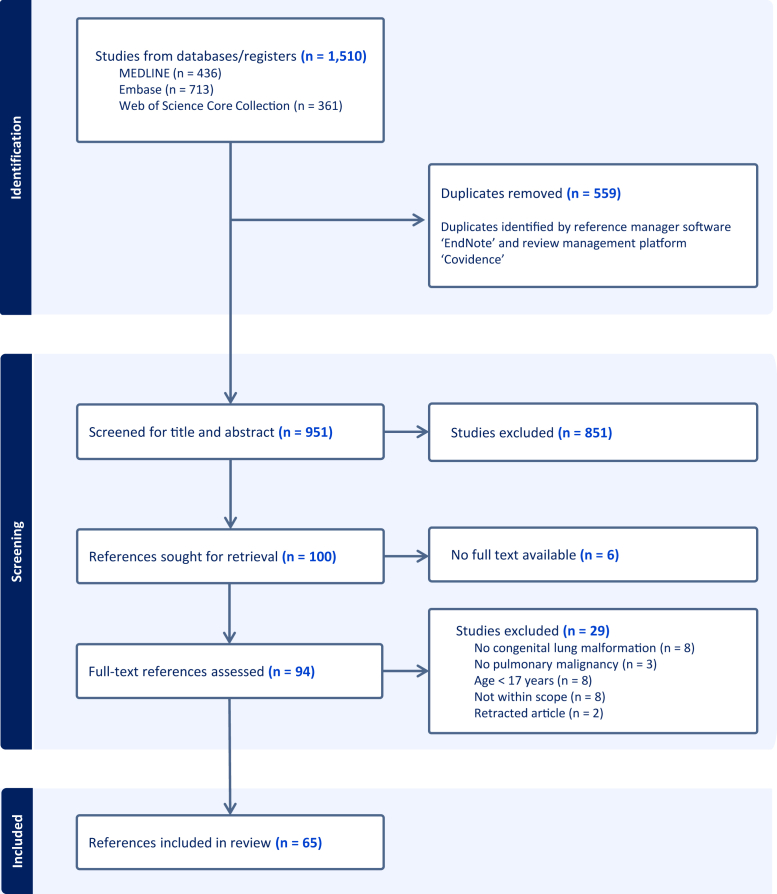
Selection of references.

A total of 1510 references were identified through searches across three databases, as outlined in Figure 1. After removing 559 duplicates, we screened 951 references based on title, abstract, and keywords, among whom 851 were excluded. One hundred references were selected for full-text retrieval, and 94 full-text references were assessed. Ultimately, 65 references were included in the review.^17^, ^18^, ^19^, ^20^, ^21^, ^22^, ^23^, ^24^, ^25^, ^26^, ^27^, ^28^, ^29^, ^30^, ^31^, ^32^, ^33^, ^34^, ^35^, ^36^, ^37^, ^38^, ^39^, ^40^, ^41^, ^42^, ^43^, ^44^, ^45^, ^46^, ^47^, ^48^, ^49^, ^50^, ^51^, ^52^, ^53^, ^54^, ^55^, ^56^, ^57^, ^58^, ^59^, ^60^, ^61^, ^62^, ^63^, ^64^, ^65^, ^66^, ^67^, ^68^, ^69^, ^70^, ^71^, ^72^, ^73^, ^74^, ^75^, ^76^, ^77^, ^78^, ^79^, ^80^, ^81^ Descriptive characteristics of the included references are presented in Table 1. Individual reference data are detailed in Supplementary File 3.

**Table 1:** Description of Included References

References Included, N	65
Patients per reference (median, range)	1 (1–17)
Publication type, N (%)	
Case report	54 (83%)
Case series	2 (3%)
Cohort study	1 (2%)
Conference abstract	8 (12%)
Region of origin, N (%)	
Europe	27 (41%)
North America	15 (23%)
Asia	21 (32%)
Oceania	1 (2%)
South America	1 (2%)
Publication year (range)	1979–2025
Period of publication, N (%)	
Before 1991	4 (6%)
1991–2000	8 (12%)
2001–2010	12 (18%)
2011–2020	28 (44%)
2021–2025	13 (20%)

### Summary of Results

A total of 85 patients with CLM and pulmonary malignancy were reported across the 65 included references. An overview of charted data of these patients is presented in Table 2.

**Table 2:** Overview of Charted Reference Data

Characteristic	Categories	Sub categories	Number of Patients (N)		Percentage (%)	Sub percentage
Sex						
	Male		43		54%	
	Female		37		46%	
	Not reported		5			
CLM type						
	CPAM		46		54%	
	ILBPS		14		16%	
	ELBPS		6		7%	
	BPS not further specified		4		5%	
	BC		15		18%	
	CLO		0		0%	
	Bronchial atresia		0		0%	
Pulmonary malignancy type						
	Adenocarcinoma		56		66%	
		Mucinous		27		48%
		Mixed		3		6%
		Reported as "BAC"		9		16%
		Not further specified		17		30%
	Carcinoid		8		9%	
	Carcinoma (other)		14		17%	
		Squamous cell		5		36%
		Mucoepidermoid		3		21%
		Other^a^		6		43%
	Non-carcinoma (other)		7		8%	
		Pulmonary blastoma		1		14%
		Sarcoma		2		29%
		Other^b^		4		57%
Age at cancer diagnosis in years (median, range)			43.5 (17–82), based on N = 80			
	Not reported		5			
Smoking status						
	Non-smoker		20		56%	
	Active smoker		13		36%	
	Former smoker		3		8%	
	Not reported		49			
Symptoms^c^						
	Asymptomatic		13		18%	
	Respiratory		48		67%	
	Systemic		13		18%	
	Pain		17		24%	
	Other^d^		5		7%	
	Not reported		13			
Treatment administered^c^						
	Resection of lesion^e^		73		94%	
		Segmentectomy		3		4%
		Lobectomy		50		68%
		Pneumonectomy		4		6%
		Non-anatomical resection		4		6%
		Other		17		23%
	Lymph node dissection		20		26%	
	Chemotherapy		13		17%	
	Radiotherapy		4		5%	
	Other^f^		5		6%	
	Not reported		7			
Follow-up status						
	Alive + no evidence of disease		32		82%	
	Alive + recurrence		1		3%	
	Died of disease		5		13%	
	Died of other cause		1		3%	
	Not reported		46			

BAC, Bronchoalveolar carcinoma, an outdated term which entails subtypes of invasive adenocarcinoma and adenocarcinoma in situ; BC, bronchogenic cyst; BPS, bronchopulmonary sequestration; CLM, congenital lung malformation; CLO, congenital lobar overinflation; CPAM, congenital pulmonary airway malformation; ELBPS, extralobar bronchopulmonary sequestration; ILBPS, intralobar bronchopulmonary sequestration.

a Other forms of carcinoma included large cell carcinoma (N = 3), lymphoepithelioma-like carcinoma (N = 1).

b Other forms of non-carcinoma malignancy included malignant pigmented perivascular epithelioid cell neoplasm (N = 1), melanoma (N = 1), mesothelioma (N = 1), and metastasis of seminoma (N = 1).

c As combinations of multiple symptom and treatment categories were possible, cumulative percentages do not amount to 100%.

d Other symptoms included vocal cord paresis (N= 1), dysphagia (N = 1), digital clubbing (N = 1), hyponatriemia (N =1), and subcutaneous mass (N = 1).

e Five patients underwent multiple surgeries.

f Other treatment included embolization (N = 2), immunotherapy (N = 2), and palliative comfort care (N = 1).

Sex was documented for 80 patients, with 43 males (54%) and 37 females (46%). CPAM was the most frequently reported CLM type, accounting for 46 cases (54%), followed by various types of BPS (including ILBPS, extralobar BPS, and unspecified BPS) in 24 cases (28%) and BC in 15 cases (18%). Among the 63 patients for whom this information was available, 21% had already been diagnosed with CLM before pulmonary malignancy detection, whereas the remaining 79% were diagnosed with CLM at the time of malignancy diagnosis.

**Figure 2:**
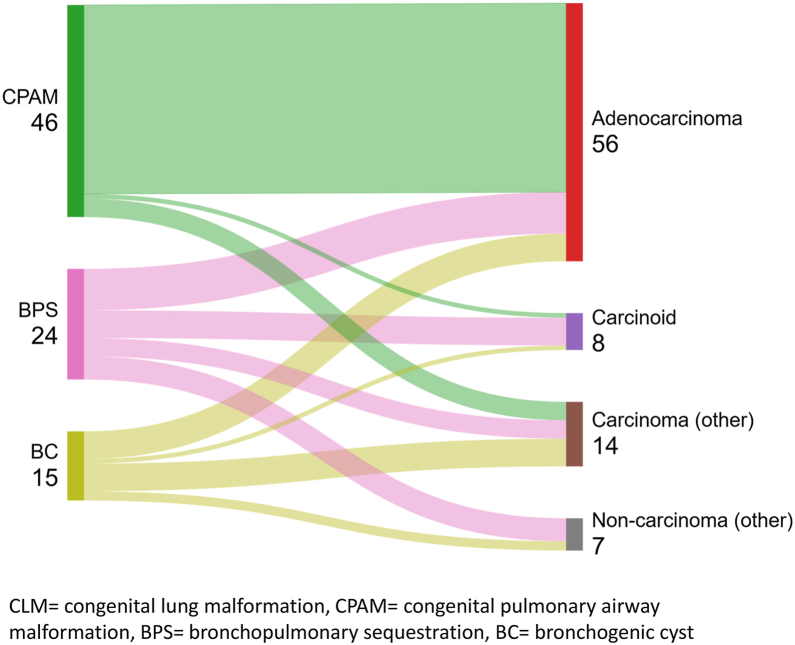
CLM type and associated malignancies. BC, bronchogenic cyst; BPS, bronchopulmonary sequestration; CLM, congenital lung malformation; CPAM, congenital pulmonary airway malformation.

Pulmonary adenocarcinoma was the most prevalent malignancy type across all CLM types, present in 56 patients (66%), with mucinous adenocarcinoma being the most common subtype (48%) (Table 2). Pulmonary adenocarcinoma was especially predominant in patients with underlying CPAM, representing 89% of malignancies in this group (Table 3, Fig. 2). No pleuropulmonary blastoma cases were identified among the included adults. TNM staging was reported for a few patients and is detailed in Table 3. The median age at cancer diagnosis was 43.5 (range 17–82) years, with no notable differences observed across malignancy types (Table 3).

**Table 3:** CLM and Associated Malignancy Data

	Malignancy Type			
	Adenocarcinoma (N = 56)	Carcinoid (N = 8)	Carcinoma (Other) (N = 14)	Non-Carcinoma (Other) (N = 7)
CLM type				
CPAM (N = 46)	41 (89%)	1 (2%)	4 (9%)	0
ILBPS (N = 14)	7 (50%)	3 (21%)	3 (21%)	1 (7%)
ELBPS (N = 6)	1 (17%)	1 (17%)	0	4 (67%)
BPS not further specified (N = 4)	1 (25%)	2 (50%)	1 (25%)	0
BC (N= 15)	6 (40%)	1 (7%)	6 (40%)	2 (13%)
TNM classification^16^ (as provided in primary references)				
Tumor	Reported (N = 30)	Reported (N = 4)	Reported (N = 5)	Reported (N = 0)
In situ	9 (30%)	-	-	-
1	6 (20%)	3 (75%)	2 (40%)	-
2	8 (27%)	1 (25%)	2 (40%)	-
3	4 (13%)	-	1 (20%)	-
4	3 (10%)	-	0	-
Nodes	Reported (N = 20)	Reported (N = 4)	Reported (N = 4)	Reported (N = 0)
0	16 (80%)	4 (100%)	3 (75%)	-
1	1 (5%)	-	-	-
2	2 (10%)	-	1 (25%)	-
3	1 (5%)	-	-	-
Metastasis	Reported (N = 21)	Reported (N = 4)	Reported (N = 4)	Reported (N = 0)
0	19 (91%)	4 (100%)	3 (75%)	-
1	2 (9%)	-	1 (25%)	-
Age at cancer diagnosis in years median (range)	42 (17–80)	41 (21–67)	51 (19–77)	46 (34–82)

N values were presented as median (%).

BC, bronchogenic cyst; BPS, Bronchopulmonary sequestration; CLM, congenital lung malformation; CPAM, congenital pulmonary airway malformation; ELBPS, extralobar bronchopulmonary sequestration; ILBPS, intralobar bronchopulmonary sequestration.

In 79 patients (93%), the malignancy arose within or in direct continuity with the CLM, whereas in six cases (7%), it developed in adjacent parenchyma of the same lung, without such continuity. No malignancies were reported in the contralateral lung.

Smoking status was reported for 36 of the 85 included patients, with data missing for 49. Among those with reported smoking data, 13 were active smokers (36%) and three were former smokers (8%). We found no statistically significant association between smoking status and malignancy type using the Fisher’s exact test (*p* = 0.71).

In total, 13 patients (18%) were asymptomatic at the time of pulmonary malignancy diagnosis. Among them, the following indications for resection were reported in 11 patients: malignancy was suspected after diagnostic workup following an incidental finding in six patients, confirmed in four, and one patient requested resection of the lesion. Most patients, 48 (67% of those with available symptom data), presented with respiratory symptoms, of whom 29 (60%) had new-onset complaints and 19 (40%) had an exacerbation of preexisting pulmonary morbidity. The most common respiratory symptoms were cough (42%), hemoptysis (31%), respiratory or lesion infections (29%), dyspnea (27%), and pneumothorax (4%).

Furthermore, 13 patients (18% of those with available symptom data) presented with systemic symptoms that included fever (61%), asthenia (31%), and weight loss (23%). Two patients experienced two systemic symptoms. Pain, primarily in the chest area, was reported by 17 patients (24%), and other symptoms were reported by five patients (7%), as described in Table 2.

Surgical treatment was the primary management approach for the detected pulmonary lesions, performed in 94% (73/78) of patients with available treatment data. Pulmonary malignancy was suspected or diagnosed before resection in 43% of patients, whereas 57% of malignancies were identified postoperatively. Among those diagnosed with malignancy postoperatively, 90% underwent surgery due to symptoms, 7% due to preoperative suspicion of malignancy, and 3% based on patient preference for surgical CLM resection. Lobectomy was the predominant type of resection, performed in for 68% (50/73) of operatively managed patients. Additional therapies were administered in fewer patients, including—mainly hilar or mediastinal—lymph node dissection (20 patients, 26%), chemotherapy (13 patients, 17%), radiotherapy (four patients, 5%), and other treatment forms (five patients, 6%), including embolization (N = 2), immunotherapy (N = 2), and palliative comfort care (N = 1).

Follow-up status was documented for 39 of 85 patients. Among these, 32 patients (82% of reported) were alive with no evidence of recurring disease with a median follow-up period of 14 (range 1–272) months, whereas five (13%) died due to malignant disease. The age at diagnosis for these five deceased patients ranged from 19 to 75 years, with survival durations of 18 to 68 months after lung cancer diagnosis.^21^^,^^24^^,^^37^^,^^48^^,^^70^

Patients who passed away due to pulmonary malignancy had adenocarcinoma (N = 3), large cell anaplastic carcinoma (N = 1), and melanoma (arising from BC) in one instance. The underlying CLM types were CPAM in two patients, BC in two patients, and ILBPS in one patient. TNM staging was available for two deceased patients, both of whom had metastatic disease (M1) secondary to lung adenocarcinoma at diagnosis.^24^^,^^48^

## Discussion

This scoping review provides a consolidated overview of reported malignancies in adults with CLM, highlighting patterns of presentation, histology, and management while addressing evidence gaps. Most patients presented symptomatically, though a meaningful subset was asymptomatic at malignancy diagnosis. Adenocarcinoma—often mucinous—predominated, particularly within CPAM. Surgery was the principal treatment, with selective use of adjuvant therapy, and more than half of malignancies were recognized only after resection. Despite incomplete follow-up, disease control and prognosis among documented cases seemed generally favorable. However, these findings should be interpreted cautiously in, as they may partially reflect the relatively early stage of discovered malignancies—including a considerable amount of in situ tumors and those with N0 nodal status—and the significant proportion of more indolent subtypes such as carcinoids within this cohort.

The observed distribution of malignancy types differs from typical patterns in the broader NSCLC population.^82^ The overrepresentation of adenocarcinoma—especially of the mucinous subtype—in CLM-associated malignancies may reflect unique biological pathways involved in malignancy development, particularly within CPAM. Adenocarcinoma in NSCLC frequently involves driver mutations in oncogenes such as *EGFR*, *KRAS*, and *BRAF*, whereas such mutations are rare in squamous cell carcinoma.^83^^,^^84^ Interestingly, comparable oncogenic mutations—mostly *KRAS*—have been identified in CPAM, indicating that these genetic alterations might contribute to malignant transformation.^85^ The predominance of mucinous adenocarcinoma in this cohort underscores the need for further investigation into the molecular mechanisms driving malignancy in this distinctive population. Notably, among patients with BPS, carcinoid tumors accounted for one-third of reported malignancies, a striking overrepresentation given that carcinoids comprise only 2% of all primary lung cancers.^86^ This finding suggests that specific factors within BPS tissue may favor neuroendocrine differentiation and tumorigenesis.

Clinical presentations of pulmonary malignancies in adults with CLM—predominantly cough and dyspnea—mirrored population-based lung cancer patterns, with a meaningful subset of asymptomatic patients.^87^ The median age at lung cancer diagnosis among the reviewed patients was 43.5 years, which is notably younger than the global median age of 70 years for lung cancer diagnosis.^88^ Combined with the relatively favorable postoperative outcomes and high proportion of never-smokers—uncommon in general lung cancer cohorts—this raises the possibility of distinct tumor biology in CLM-associated malignancies. The observation that nearly all malignancies arose within or in direct continuity with the CLM further supports the notion that these tumors represent malignant transformation of preexisting congenital tissue rather than coincidental de novo lung cancers.

Interestingly, more than half (57%) of all pulmonary malignancy diagnoses in this cohort were established only after surgical resection of the lung lesion, emphasizing that imaging, cytology, and biopsy do not always provide a definitive conclusion in the presence of a pulmonary malignancy, while also demonstrating that pulmonary malignancies can closely resemble CLM on medical imaging, complicating preoperative diagnosis.

Although most included cases involved patients in whom the CLM was first identified at the time of malignancy diagnosis, the predominance of symptom-driven resections highlights the need for renewed evaluation when new or worsening complaints arise in adults with known CLM, as such changes may signal malignant transformation. The presence of malignancies in asymptomatic patients, the younger median age at diagnosis, and the relatively high proportion of malignancy diagnoses established postoperatively further underscore the importance of proactive and tailored surveillance strategies in adults with known CLM—particularly prenatally detected cohorts now transitioning to adult care. The absence of symptoms among individuals with known CLM should not lead to complacency in evaluations and follow-up. Routine imaging, such as chest computed tomography scans, and comprehensive assessments remain critical for the early detection of malignancies. In light of the ongoing debate on the optimal management of asymptomatic patients with CLM, future research should focus on refining imaging and follow-up protocols, developing biomarker strategies to enhance malignancy risk prediction, and identifying subgroups who may potentially benefit from prophylactic CLM resection. To support this agenda, a multidisciplinary Task Force is currently developing an evidence-based clinical practice guideline on diagnostic imaging in CLM with the support of the European Respiratory Society.

A recently published review by Pederiva et al.^89^ deserves commendation for drawing attention to the long-term oncological burden of CLM and advocates universal CLM resection. However, the statistical modeling treated CLM subtype as an outcome and downstream events (including tumor status) as predictors, a reverse temporality that can yield biologically implausible interpretations. In addition, prevalence claims such as “74% of adults with CLM eventually develop symptoms” and “11% of CLM harbor lung tumors” derive from literature dominated by case reports and series that are highly vulnerable to publication and reporting bias, likely overstating population risk. Accordingly, such figures should be treated as prompts for further investigation rather than grounds for changing current practice.

Several limitations of this scoping review are also worth addressing. First, a reliable incidence of malignant transformation in adults with CLM could not be established because the underlying prevalence of CLM in adults remains unknown. Second, the included literature mainly consisted of case reports and case series, which are prone to reporting bias and limited generalizability. Reporting bias could have led to an emphasis on unusually young patients with positive outcomes, leaving older patients with less favorable results underrepresented. In addition, small sample sizes and descriptive data limit the broader applicability of findings. As we did not formally perform a risk-of-bias assessment for included references, all numerical outputs—including the median age at malignancy—are to be considered as hypothesis-generating results rather than definitive effect estimates.

Third, the retrospective nature of the included studies complicates the application of contemporary classifications of malignancies. For example, the outdated term bronchoalveolar carcinoma (BAC) was reported in multiple older cases.^21^^,^^28^^,^^30^^,^^51^^,^^60^^,^^61^^,^^68^^,^^74^ In the 2015 and 2021 World Health Organization reclassifications of lung adenocarcinoma, the term BAC was replaced by the introduction of concepts such as “adenocarcinoma in situ,” “minimally invasive adenocarcinoma,” and “invasive lepidic adenocarcinoma.”^90^^,^^91^ As retrospective reclassification was unfeasible, malignancies originally described as BAC were classified under a specific adenocarcinoma subgroup. Although outdated terminology presents interpretative challenges, its inclusion provides important historical context and highlights the evolution of classification systems.

Last, there was significant heterogeneity across studies in the reporting of smoking status, treatments, outcomes, and follow-up data. For example, although most studies reported the surgical procedures performed, data on adjunct therapies and long-term outcomes were often incomplete or inconsistently reported. Consequently, drawing definitive conclusions on these topics was challenging. However, this review highlights the importance of standardized reporting of treatment and outcomes, which should be a focus of future research.

Despite these limitations, this scoping review provides a comprehensive synthesis of the available clinical evidence on CLM-associated pulmonary malignancies. It underscores the need for standardized reporting and large prospective registries to clarify incidence and identify adults with CLM at highest risk of malignant transformation. In addition, further research into the biological and molecular pathways underlying malignant transformation is crucial for improving risk assessment and identifying patient subgroups at higher risk of developing malignancies.

## CRediT Authorship Contribution Statement

**Louis W. J. R. Dossche**: Conceptualization, Methodology, Data curation, Investigation, Formal analysis, Writing – original draft, Writing – review & editing.

**M. H. T. Heuff**: Data curation, Investigation, Formal analysis, Writing – review & editing.

**A. M. Heijne den Bak**: Formal analysis, Writing – review & editing.

**A. C. Dingemans**: Formal analysis, Writing – review & editing.

**L. S. Kamphuis**: Conceptualization, Methodology, Data curation, Investigation, Formal analysis, Writing – original draft, Writing – review & editing.

**J. Marco Schnater**: Conceptualization, Methodology, Data curation, Investigation, Formal analysis, Writing – original draft, Writing – review & editing.

All authors have read and approved the final manuscript version for publication and agree to be accountable for all aspects of the work, ensuring questions related to the accuracy and integrity of any part of the work are appropriately investigated and resolved.

## Data Sharing Statement

All extracted, individual-reference-level data from this scoping review are publicly available as Supplementary File 3, accessible on publication. Any additional data or related documents will be available on reasonable request from other researchers by contacting the corresponding author via e-mail at j.schnater@erasmusmc.nl. Data will be shared following approval of the proposed analyses and a signed data access agreement.

## Disclosure

Prof. Dingemans reports receiving institutional grants and fees from multiple pharmaceutical companies, all unrelated to this article. The remaining authors declare no conflict of interest.
