# Supplementary material for: Pulmonary Malignancies in Adults With Congenital Lung Malformations: A Scoping Review
Source: JTO Clin Res Rep. 2026 Jan 16;7(4):100958. doi: 10.1016/j.jtocrr.2026.100958 (PMC12992512; doi:10.1016/j.jtocrr.2026.100958)
Supplement: Supplementary Files 1-3 [file mmc1.pdf]

Supplemental File 1.

Preferred Reporting Items for Systematic reviews and Meta-Analyses extension for Scoping Reviews (PRISMA-ScR) Checklist

SECTION	ITEM	PRISMA-ScR CHECKLIST ITEM	REPORTED ON PAGE #
**TITLE**			
Title	1	Identify the report as a scoping review.	1
**ABSTRACT**			
Structured summary	2	Provide a structured summary that includes (as applicable): background, objectives, eligibility criteria, sources of evidence, charting methods, results, and conclusions that relate to the review questions and objectives.	2
**INTRODUCTION**			
Rationale	3	Describe the rationale for the review in the context of what is already known. Explain why the review questions/objectives lend themselves to a scoping review approach.	3
Objectives	4	Provide an explicit statement of the questions and objectives being addressed with reference to their key elements (e.g., population or participants, concepts, and context) or other relevant key elements used to conceptualize the review questions and/or objectives.	4
**METHODS**			
Protocol and registration	5	Indicate whether a review protocol exists; state if and where it can be accessed (e.g., a Web address); and if available, provide registration information, including the registration number.	5
Eligibility criteria	6	Specify characteristics of the sources of evidence used as eligibility criteria (e.g., years considered, language, and publication status), and provide a rationale.	5
Information sources*	7	Describe all information sources in the search (e.g., databases with dates of coverage and contact with authors to identify additional sources), as well as the date the most recent search was executed.	5
Search	8	Present the full electronic search strategy for at least 1 database, including any limits used, such that it could be repeated.	Suppl. 2
Selection of sources of evidence†	9	State the process for selecting sources of evidence (i.e., screening and eligibility) included in the scoping review.	5
Data charting process‡	10	Describe the methods of charting data from the included sources of evidence (e.g., calibrated forms or forms that have been tested by the team before their use, and whether data charting was done independently or in duplicate) and any processes for obtaining and confirming data from investigators.	5-6
Data items	11	List and define all variables for which data were sought and any assumptions and simplifications made.	6
Critical appraisal of individual sources of evidence§	12	If done, provide a rationale for conducting a critical appraisal of included sources of evidence; describe the methods used and how this information was used in any data synthesis (if appropriate).	N/a
Synthesis of results	13	Describe the methods of handling and summarizing the data that were charted.	6

St. Michael's
Inspired Care.
Inspiring Science.

SECTION	ITEM	PRISMA-ScR CHECKLIST ITEM	REPORTED ON PAGE #
**RESULTS**			
Selection of sources of evidence	14	Give numbers of sources of evidence screened, assessed for eligibility, and included in the review, with reasons for exclusions at each stage, ideally using a flow diagram.	Fig. 1
Characteristics of sources of evidence	15	For each source of evidence, present characteristics for which data were charted and provide the citations.	Suppl. 3
Critical appraisal within sources of evidence	16	If done, present data on critical appraisal of included sources of evidence (see item 12).	N/a
Results of individual sources of evidence	17	For each included source of evidence, present the relevant data that were charted that relate to the review questions and objectives.	Suppl. 3
Synthesis of results	18	Summarize and/or present the charting results as they relate to the review questions and objectives.	T2, T3, Fig. 2
**DISCUSSION**			
Summary of evidence	19	Summarize the main results (including an overview of concepts, themes, and types of evidence available), link to the review questions and objectives, and consider the relevance to key groups.	13
Limitations	20	Discuss the limitations of the scoping review process.	15
Conclusions	21	Provide a general interpretation of the results with respect to the review questions and objectives, as well as potential implications and/or next steps.	15
**FUNDING**			
Funding	22	Describe sources of funding for the included sources of evidence, as well as sources of funding for the scoping review. Describe the role of the funders of the scoping review.	1

JBI = Joanna Briggs Institute; PRISMA-ScR = Preferred Reporting Items for Systematic reviews and Meta-Analyses extension for Scoping Reviews.
* Where *sources of evidence* (see second footnote) are compiled from, such as bibliographic databases, social media platforms, and Web sites.
† A more inclusive/heterogeneous term used to account for the different types of evidence or data sources (e.g., quantitative and/or qualitative research, expert opinion, and policy documents) that may be eligible in a scoping review as opposed to only studies. This is not to be confused with *information sources* (see first footnote).
‡ The frameworks by Arksey and O'Malley (6) and Levac and colleagues (7) and the JBI guidance (4, 5) refer to the process of data extraction in a scoping review as data charting.
§ The process of systematically examining research evidence to assess its validity, results, and relevance before using it to inform a decision. This term is used for items 12 and 19 instead of "risk of bias" (which is more applicable to systematic reviews of interventions) to include and acknowledge the various sources of evidence that may be used in a scoping review (e.g., quantitative and/or qualitative research, expert opinion, and policy document).

From: Tricco AC, Lillie E, Zarin W, O'Brien KK, Colquhoun H, Levac D, et al. PRISMA Extension for Scoping Reviews (PRISMAScR): Checklist and Explanation. Ann Intern Med. 2018;169:467–473. doi: 10.7326/M18-0850.

Supplemental File 2. Search queries

Embase search query

Period covered: 1971 – January 2025

(‘congenital lung malformation’/de OR ‘lung malformation’/de OR ‘cystic adenomatoid malformation’/de OR ‘congenital pulmonary airway malformation’/de OR ‘lung sequestration’/de OR (((congenital*) NEAR/6 (lung* OR pulmonar*) NEAR/6 (malform* OR abnormal* OR anomal*)) OR ((cystic) NEAR/6 (adenoma*) NEAR/6 (malform*)) OR CPAM OR ((congenital) NEAR/6 (pulmon* OR lung*) NEAR/6 (airway*) NEAR/6 (malform*)) OR ((lung* OR pulmonar* OR bronchopulmonar*) NEAR/3 (sequestrat*)) OR ((bronchogenic*) NEAR/3 (cyst*))):ab,ti,kw) **AND** (‘lung cancer’/exp OR (((lung* OR pulmonar* OR pleuropulm*) NEAR/3 (cancer* OR tumor* OR tumour* OR malign* OR carcinom* OR neoplas* OR blastom* OR adenocarcinom*))):ab,ti,kw) AND [ENGLISH]/lim NOT ('editorial'/de) NOT (note/de)

Medline search query

Period covered: 1946 – January 2025

(Cystic Adenomatoid Malformation of Lung, Congenital/ OR Respiratory System Abnormalities/ OR Bronchopulmonary Sequestration/ OR (((congenital*) ADJ6 (lung* OR pulmonar*) ADJ6 (malform* OR abnormal* OR anomal*)) OR ((cystic) ADJ6 (adenoma*) ADJ6 (malform*)) OR CPAM OR ((congenital) ADJ6 (pulmon* OR lung*) ADJ6 (airway*) ADJ6 (malform*)) OR ((lung* OR pulmonar* OR bronchopulmonar*) ADJ3 (sequestrat*)) OR ((bronchogenic*) ADJ3 (cyst*))).ab,ti,kf.) **AND** (exp Lung Neoplasms/ OR (((lung* OR pulmonar* OR pleuropulm*) ADJ3 (cancer* OR tumor* OR tumour* OR malign* OR carcinom* OR neoplas* OR blastom* OR adenocarcinom*))).ab,ti,kw.) AND english.la. NOT (editorial).pt. NOT (Comment.pt.)

Web of Science search query

Period covered: 1975 – January 2025

TS=(((((congenital*) NEAR/5 (lung* OR pulmonar*) NEAR/5 (malform* OR abnormal* OR anomal*)) OR ((cystic) NEAR/5 (adenoma*) NEAR/5 (malform*)) OR CPAM OR ((congenital) NEAR/5 (pulmon* OR lung*) NEAR/5 (airway*) NEAR/5 (malform*)) OR ((lung* OR pulmonar* OR bronchopulmonar*) NEAR/2 (sequestrat*)) OR ((bronchogenic*) NEAR/2 (cyst*)))) **AND** ((((lung* OR pulmonar* OR pleuropulm*) NEAR/2 (cancer* OR tumor* OR tumour* OR malign* OR carcinom* OR neoplas* OR blastom* OR adenocarcinom*))))) AND LA=(English) NOT DT=(Editorial Material) NOT DT=(Note)

Supplemental File 3. Individual reference info

First author and year	Publication year	Publication type	Region of origin	Number of patients	Sex	CLM type
Akinsanya et al. 2025	2025	Case report	North America	1	Female	BC
Aribindi et al. 2024	2024	Conference abstract	North America	1	Female	CPAM
Ashizawa et al. 2001	2001	Case report	Asia	1	Male	BC
Belchis et al. 2014	2014	Case report	North America	1	Male	ELBPS
Bell-Thomson et al	1979	Case report	North America	1	Male	ILBPS
Benjamin et al. 1991	1991	Case report	North America	1	Male	CPAM
Benouaich et al. 2009	2009	Case report	Europe	1	Male	CPAM
Calzada et al. 2011	2011	Case report	North America	1	Female	BC
Chang et al. 2021	2021	Case series	Europe	17	Male	CPAM
					Male	CPAM
					Female	CPAM
					Female	CPAM
					Female	CPAM
					Female	CPAM
					Male	CPAM
					Male	CPAM
					Female	CPAM
					Female	CPAM
					Male	CPAM
					Male	CPAM
					Male	CPAM
					Male	CPAM
					Male	CPAM
					Female	CPAM
					Male	CPAM
Cuypers et al. 1996	1996	Case series	Europe	1	N/A	BC
Daddi et al. 2021	2021	Conference abstract	Europe	1	Male	CPAM
Daley et al. 2023	2023	Conference abstract	North America	1	Female	CPAM
de Perrot et al. 2001	2001	Case report	Europe	1	Male	BC
Di Crescenzo et al. 2013	2013	Case report	Europe	1	Female	ELBPS
Endo et al. 2000	2000	Case report	Asia	1	Female	BC
Eustace et al. 1996	1996	Case report	North America	1	Male	BPS
Fiorelli et al. 2012	2012	Case report	Europe	1	Female	BC
Frick et al. 2021	2021	Case report	Europe	1	Female	CPAM
Frick et al. 2021	2016	Case report	Europe	1	Male	CPAM
Fudulu et al. 2016	2017	Case report	Europe	1	Male	ELBPS
Gatzinsky et al. 1988	1988	Case report	Europe	1	Female	ILBPS

Gomez-Hernandez et al. 2017	2017	Case report	Europe	1	Female	BC
Granato et al. 2009	2009	Case series	Europe	1	Male	BC
Harini et al. 2012	2012	Case report	Asia	1	Male	CPAM
Hasegawa et al. 2014	2014	Case report	Asia	1	Male	CPAM
He et al. 2023	2023	Case report	Asia	1	Female	ILBPS
Hekelaar et al. 2000	2000	Case report	Europe	1	Female	ILBPS
Irugulapati et al. 2012	2012	Conference abstract	North America	1	Female	CPAM
Iskandir et al. 2021	2021	Conference abstract	North America	1	Female	BPS
Jakopovic et al. 2005	2005	Case report	Europe	1	Female	BC
Juettner et al. 1985	1985	Case report	Europe	1	Male	ILBPS
Kimura et al. 2020	2020	Case report	Asia	1	Female	BC
Lantuejoul et al. 2006	2006	Case report	Europe	2	Male	CPAM
					Male	CPAM
Lawal et al. 2011	2011	Case report	Europe	1	Male	ILBPS
Long et al. 2019	2019	Conference abstract	North America	1	Male	CPAM
Mani et al. 2007	2007	Case report	North America	1	Female	CPAM
Maskey et al. 2011	2011	Conference abstract	Europe	1	Male	CPAM
Mengoli et al. 2016	2016	Case report	Europe	1	Male	ELBPS
Muhammad et al. 2022	2022	Case report	Asia	1	Female	ILBPS
Nakanishi et al. 2021	2021	Case report	Asia	1	Female	ILBPS
Nowak et al. 2013	2013	Case report	Europe	1	Male	ILBPS
O'Dell et al. 2016	2016	Case report	North America	1	Male	BPS
Okamoto et al. 2005	2005	Case report	Asia	1	Male	ILBPS
Paksoy et al. 1992	1992	Case report	Asia	1	Female	ILBPS
Pelosi et al. 2019	2019	Case report	Europe	1	Female	CPAM
Rao et al. 2011	2011	Conference abstract	North America	1	Male	CPAM
Ribet et al. 1995	1995	Case report	Europe	1	Female	CPAM
Santosham et al. 2019	2019	Case report	Asia	1	Male	CPAM
Sato et al. 2016	2016	Case report	Asia	1	Female	ELBPS
Senturk et al. 2010	2010	Case report	Asia	1	Female	ELBPS
Sheffield et al. 1987	1987	Case report	Europe	1	Male	CPAM
Singh et al. 2016	2016	Case report	North America	1	Female	CPAM
Soler-Perromat et al. 2019	2019	Case report	Europe	1	Female	CPAM
Sudou et al. 2003	2003	Case report	Asia	1	Male	CPAM
Taira et al. 2018	2018	Case report	Asia	1	Female	BC
Tanita et al. 22	2002	Case report	Asia	1	Male	BC
Tsai et al. 2012	2012	Case report	Asia	1	Female	BC
Usui et al. 1991	1991	Case report	Asia	1	Male	CPAM
Wang et al. 2013	2013	Case report	Oceania	1	Male	ILBPS
West et al. 2007	2007	Case report	Europe	1	Male	CPAM
Westphal et al. 2012	2012	Case report	South America	1	Female	ILBPS
Whooley et al. 2022	2022	Case report	Europe	1	Male	BC
Xu et al. 2018	2018	Case report	Asia	1	Female	BPS

Yarlagadda et al. 2024	2024	Case report	Asia	1	Male	ILBPS
Zeng et al. 2022	2022	Cohort study	Asia	4	N/A	CPAM
					N/A	CPAM
					N/A	CPAM
					N/A	CPAM
				85		

CLM= congenital lung malformation, CPAM= congenital pulmonary airway malformation, ILBPS= intralobar bronchopulmonary sequestration, ELBPS= extralobar bronchopulmonary seques

Type of malignancy	Tumor (TNM)	Nodal (TNM)	Metastasis (TNM)	Age at cancer diagnosis	Smoking status
Adenosquamous carcinoma	3	0	0	74	Never
Adenocarcinoma (not further specified)	N/A	N/A	N/A	44	Non-smoker
Adenocarcinoma (not further specified)	N/A	N/A	N/A	42	N/A
Adenocarcinoma (not further specified)	2	0	0	70	Smoker
Squamous cell carcinoma	N/A	N/A	N/A	69	Ex-smoker
"BAC" (neoplastic, well-differentiated mucus-secreting cells in alveolar walls)	N/A	N/A	N/A	19	Never
Adenocarcinoma (mixed)	N/A	N/A	N/A	77	N/A
Adenocarcinoma (not further specified)	N/A	N/A	N/A	32	N/A
Mucinous adenocarcinoom	0	0	0	17	N/A
Mucinous adenocarcinoom	1	N/A	N/A	18	N/A
Mucinous adenocarcinoom	3	N/A	N/A	22	N/A
Mucinous adenocarcinoom	1	N/A	N/A	27	N/A
Mucinous adenocarcinoom	0	N/A	N/A	28	N/A
Mucinous adenocarcinoom	1	N/A	N/A	30	N/A
Mucinous adenocarcinoom	0	N/A	N/A	34	N/A
Mucinous adenocarcinoom	3	N/A	1	35	N/A
Mucinous adenocarcinoom	0	N/A	N/A	35	N/A
Mucinous adenocarcinoom	0	0	0	41	N/A
Mucinous adenocarcinoom	4	0	0	42	N/A
Mucinous adenocarcinoom	0	N/A	0	51	N/A
Mucinous adenocarcinoom	4	0	0	56	N/A
Mucinous adenocarcinoom	2	0	0	57	N/A
Mucinous adenocarcinoom	0	N/A	N/A	61	N/A
Mucinous adenocarcinoom	0	N/A	N/A	64	N/A
Mucinous adenocarcinoom	2	0	0	68	N/A
Squamous cell carcinoma	N/A	N/A	N/A	70	N/A
Sarcomatoid carcinoma	1	0	0	52	Ex-smoker
Mucinous adenocarcinoma	N/A	N/A	N/A	32	Smoker
"BAC"	N/A	N/A	N/A	75	Non-smoker
Pulmonary fibrosarcoma	N/A	N/A	N/A	35	N/A
"BAC" (well-differentiated, mucus-secreting columnar cells lined the intact alveolar septa and the cyst wall)	N/A	N/A	N/A	37	Never smoker
Carcinoid	N/A	N/A	N/A	29	N/A
Adenocarcinoma (not further specified)	1	0	0	65	N/A
Squamous cell carcinoma	1	N/A	N/A	47	Smoker (20 pack years)
Mucinous adenocarcinoma	N/A	N/A	N/A	68	N/A
Testicular seminoma (pulmonary metastasis)	N/A	N/A	N/A	46	N/A
Adenocarcinoma (not further specified)	N/A	N/A	N/A	50	Smoker

Mucinous adenocarcinoma	N/A	N/A	N/A	76	Non-smoker
Large-cell anaplastic carcinoma	N/A	N/A	N/A	75	Smoker
Mucoepidermoid carcinoma	N/A	N/A	N/A	19	N/A
Adenocarcinoma (not further specified)	2	0	0	80	Smoker
Adenocarcinoma (not further specified)	2	2	0	52	Non-smoker
Lymphoeputhelioma-like carcinoma	2	0	0	31	Non-smoker
Mucinous adenocarcinoma	N/A	1	N/A	43	Non-smoker
Carcinoid	N/A	N/A	N/A	21	N/A
Large-cell carcinoma	N/A	N/A	N/A	40	N/A
Carcinoid	2	0	0	45	Non-smoker
Leiomyosarcoma	N/A	N/A	N/A	82	N/A
Adenocarcinoma (mixed)	N/A	N/A	N/A	60	Smoker
"BAC" (of mucinous subtype)	N/A	N/A	N/A	32	N/A
Adenocarcinoma (not further specified)	1	0	1	67	Smoker (45 pack years)
Mucinous adenocarcinoma	N/A	N/A	N/A	56	Non-smoker
Adenocarcinoma (mixed)	N/A	N/A	N/A	29	N/A
"BAC" (of mucinous subtype)	N/A	N/A	N/A	51	Never smoker
Malignant pigmented perivascular epithelioid cell neoplasm	N/A	N/A	N/A	34	Never smoker
Adenocarcinoma (not further specified)	4	3	0	38	N/A
Adenocarcinoma (not further specified)	N/A	N/A	N/A	68	N/A
Carcinoid	1	0	0	41	Occasional (2 pack years)
Adenocarcinoma (not further specified)	N/A	N/A	N/A	68	N/A
Adenocarcinoma (not further specified)	1	0	0	69	Smoker (45 pack years)
Fibrous mesothelioma	N/A	N/A	N/A	64	N/A
Adenocarcinoma (not further specified)	3	2	0	42	Non-smoker
"BAC" (micropapillary, acinar and lepidic growth patterns)	N/A	N/A	N/A	18	Non-smoker
"BAC" (tubular architecture and made of mucus-secreting, unistratified or multi- stratified columnar epithelium)	N/A	N/A	N/A	42	Smoker (15-20 pack years
Mucinous adenocarcinoma (in situ)	0	0	0	28	N/A
Carcinoid	1	0	0	67	Never smoker
Pulmonary blastoma	N/A	N/A	N/A	45	Non-smoker
Adenocarcinoma (not further specified)	N/A	N/A	N/A	18	N/A
Mucinous adenocarcinoma	3	0	0	18	N/A
Mucinous adenocarcinoma	N/A	N/A	N/A	29	Ex-smoker
"BAC" (well-differentiated)	2	0	0	17	N/A
Mucoepidermoid carcinoma	N/A	N/A	N/A	77	Never smoker
Melanoma	N/A	N/A	N/A	46	N/A
Carcinoid	N/A	N/A	N/A	41	N/A
Squamous cell carcinoma	N/A	N/A	N/A	45	Smoker (56 pack years)
Adenocarcinoma (not further specified)	2	0	0	65	N/A
"BAC" (of mucinous subtype)	2	0	0	18	Non-smoker
Carcinoid	1	0	0	39	N/A
Squamous cell carcinoma	N/A	N/A	N/A	70s	Non-smoker
Large-cell lung carcinoma	2	2	1	51	N/A

Mucoepidermoid carcinoma	N/A	N/A	N/A	40	smoker
Mucinous adenocarcinoom	N/A	N/A	N/A	N/A	N/A
Mucinous adenocarcinoom	N/A	N/A	N/A	N/A	N/A
Adenocarcinoma (not further specified)	N/A	N/A	N/A	N/A	N/A
Carcinoid	N/A	N/A	N/A	N/A	N/A

stration, BPS= bronchopulmonary sequestration, BC= bronchogenic cyst, BAC= bronchoalvealar carcinoma, N/A= not available, LRTI= lower respiratory tract infection, URTI= upper res

Symptoms	Treatment	Follow-up
Hyponatriemia	Lobectomy + lymph node dissection + adjuvant chemotherapy + adjuvant immunotherapy	NED 2.4y
Cough, dyspnea on exertion, chest pain, weight loss, anorexia	Chemotherapy + immunotherapy	N/A
Asymptomatic	Resection	N/A
Chest pain	Lobectomy	N/A
Asymptomatic	Lobectomy	NED 5y after surgery
Cough	Pneumectomy + chemotherapy	Died 4y after surgery due to recurrence
Fever, cough	Lobectomy	NED 3y after surgery
Neck mass, vocal cord paresis, throat pain	Subtotal resection + chemoradiation	N/A
N/A	Non-anatomical resection	N/A
Cyst infection	Lobectomy	Recurrence after 7y NED 272m after lobectomy
N/A	Lobectomy	N/A
Recurrent LRTI	Lobectomy	N/A
N/A	Lobectomy	N/A
N/A	Lobectomy	N/A
N/A	Lobectomy	N/A
Cyst infection	Conservative (M1a)	DoD (68m)
N/A	Lobectomy	N/A
Flu-like, chest pain	Lobectomy	N/A
Cough, chest pain	Lobectomy	NED (1m)
Pneumothorax	Lobectomy	N/A
Asymptomatic	Lobectomy	NED (46m)
dyspnea	Lobectomy	NED (115m)
infections	Lobectomy	NED (10m)
N/A	Non-anatomical resection	N/A
Cough, chest pain	Lobectomy	Died unknown cause (130m)
N/A	N/A	N/A
Cough	1. Wedge resection 2. Lobectomy with lymphadenectomy	NED (6m)
Dyspnea, cough, back pain	Lobectomy	N/A
Recurrent LRTI	Lobectomy, lymphatic dissection	NED (18m)
Dyspnea, cough, hemoptysis	Pneumonectomy	N/A
Asymptomatic	Lobectomy	NED (1y)
Recurrent URTI	Resection	N/A
Chest pain	Resection	NED (unknow duration)
Asymptomatic	Lobectomy + lymphadenectomy	NED (391 days)
Recurrent LRTI, chest pain	Lobectomy	N/A
N/A	Sequestrectomy	NED (2y post metastectomy)
Cough, chest pain	Lobectomy + lymphadenectomy	N/A

Asymptomatic	Lobectomy + lymphadenectomy	NED (6m)
Dysphagia	Cystectomy	DoMD 2y
Cough, hemoptysis	Pneumectomy + lymph node dissection	NED (1y)
Asymptomatic	Lobectomy + hilomediastinal lymphadenectomy	N/A
Asymptomatic	1. Lobectomy + systemic lymphadenectomy 2. Adjuvant chemotherapy	NED (15m)
Digital clubbing, cough	1. Segmentectomy + lymphadenectomy 2. Pneumectomy	NED 4y after pneumectomy
Recurrent pneumonia, cough	Lobectomy + lymphadenectomy	N/A
Hemoptysis	Lobectomy	NED (unknow duration)
Pneumothorax, dyspnea	Lobectomy + lymphadenectomy	NED (3m)
Recurrent LRTI, hemoptysis	Lobectomy + lymphadenectomy	NED (7y)
Chest pain	Cystectomy	Recurrence, 24m after surgery
Hemoptysis	Lobectomy	N/A
Recurrent infections, hemoptysis	N/A	N/A
Recurrent LRTI, hemoptysis	Lobectomy, adjuvant chemotherapy, radiotherapy	DoD 28m after lobectomy
Recurrent LRTI	Lobectomy	N/A
Asthma	N/A	N/A
URTI, chest pain	Bullectomy	N/A
Hemoptysis	Sequestrectomy	NED (5m)
Hemoptysis, dyspnea, weight loss	(neoadjuvant) chemotherapy	N/A
Asymptomatic	Embolization + bi-lobectomy + lymphadenectomy	N/A
Hemoptysis	Lobectomy	N/A
Dyspnea, hemoptysis	Embolization (inoperable tumore)	N/A
Dyspnea	Neoadjuvant chemoradiotherapy. Lobectomy + lymphadenectomy	NED (1y)
Chest pain, limb pain, dyspnea, fatigue, cough	Resection	N/A
Hemoptysis	Bi-lobectomy + lymphadenectomy. Adjuvant chemotherapy	NED (8m)
Cough, hemoptysis, weight loss	Chemotherapy	N/A
Asymptomatic	segmentectomy + lymphadenectomy	NED (22m)
Cough, fever	Lobectomy	N/A
Asymptomatic	Sequestrectomy	NED (2m)
Chest pain, asthenia, cough, dyspnea, headache	Sequestrectomy + chemotherapy	NED (10m)
Hemoptysis	Resection	N/A
Recurrent infections, dyspnea	1. segmentectomy 2. lobectomy	NED (5y after lobectomy)
Fever, cough, chest pain	Lobectomy	N/A
Dyspnea on exertion	Lobectomy	NED (3m)
Chest pain	Wedge resection + partial diaphragmectomy	N/A
Left scapular mass growth	Cystectomy + chemotherapy	Died of metastasis (18m)
Chest pain	Thymectomy	NED (1m)
Hemoptysis	Lobectomy	N/A
Asymptomatic	Lobectomy	N/A
Cough, fever	Lobectomy	NED (3y)
Cough, fever	Sequestrectomy	N/A
Asymptomatic	Cystectomy + lobectomy	N/A
Fever, chest pain	Lobectomy + lymphadenectomy + geftinib	NED (31m)

Cough, fever	Bi-lobectomy	N/A
N/A	N/A	N/A
N/A	N/A	N/A
N/A	N/A	N/A
N/A	N/A	N/A

piratory tract infection, NED= no evidence of disease, DoD= died of disease
